# Variants in calcium voltage-gated channel subunit Alpha1 C-gene (*CACNA1C*) are associated with sleep latency in infants

**DOI:** 10.1371/journal.pone.0180652

**Published:** 2017-08-09

**Authors:** Katri Kantojärvi, Johanna Liuhanen, Outi Saarenpää-Heikkilä, Anna-Liisa Satomaa, Anneli Kylliäinen, Pirjo Pölkki, Julia Jaatela, Auli Toivola, Lili Milani, Sari-Leena Himanen, Tarja Porkka-Heiskanen, Juulia Paavonen, Tiina Paunio

**Affiliations:** 1 Genomics and Biomarkers Unit, National Institute for Health and Welfare, Helsinki, Finland; 2 Department of Psychiatry, University of Helsinki and Helsinki University Central Hospital, Helsinki, Finland; 3 Human Information Processing–laboratory, University of Tampere, Tampere, Finland; 4 Department of Clinical Neurophysiology, Tampere University Hospital, Medical Imaging Centre and Hospital Pharmacy, Pirkanmaa Hospital District, Tampere, Finland; 5 School of Social Sciences and Humanities/Psychology, University of Tampere, Tampere, Finland; 6 Department of Social Sciences, University of Eastern Finland, Kuopio, Finland; 7 The Estonian Genome Center, University of Tartu, Tartu, Estonia; 8 Faculty of Medicine and Life Sciences, University of Tampere, Tampere, Finland; 9 Institute of Biomedicine/Physiology, University of Helsinki, Helsinki, Finland; 10 Child and Adolescent Mental Health, National Institute for Health and Welfare, Helsinki, Finland; University of Queensland, AUSTRALIA

## Abstract

Genetic variants in *CACNA1C* (calcium voltage-gated channel subunit alpha1 C) are associated with bipolar disorder and schizophrenia where sleep disturbances are common. In an experimental model, Cacna1c has been found to modulate the electrophysiological architecture of sleep. There are strong genetic influences for consolidation of sleep in infancy, but only a few studies have thus far researched the genetic factors underlying the process. We hypothesized that genetic variants in *CACNA1C* affect the regulation of sleep in early development. Seven variants that were earlier associated (genome-wide significantly) with psychiatric disorders at *CACNA1C* were selected for analyses. The study sample consists of 1086 infants (520 girls and 566 boys) from the Finnish CHILD-SLEEP birth cohort (genotyped by Illumina Infinium PsychArray BeadChip). Sleep length, latency, and nightly awakenings were reported by the parents of the infants with a home-delivered questionnaire at 8 months of age. The genetic influence of *CACNA1C* variants on sleep in infants was examined by using PLINK software. Three of the examined *CACNA1C* variants, rs4765913, rs4765914, and rs2239063, were associated with sleep latency (permuted P<0.05). There was no significant association between studied variants and night awakenings or sleep duration. *CACNA1C* variants for psychiatric disorders were found to be associated with long sleep latency among 8-month-old infants. It remains to be clarified whether the findings refer to defective regulation of sleep, or to distractibility of sleep under external influences.

## Introduction

*CACNA1C* encodes the alpha subunit of the L-type voltage-dependent calcium channel Cav1.2, which is highly expressed in hippocampus, cerebral cortex, and cerebellum [1]. Until now, genome-wide association studies have detected seven variants at *CACNA1C* which are associated with psychiatric disorders. The Psychiatric GWAS Consortium Bipolar Disorder Working Group [2] reported genome-wide, significant association between *CACNA1C* variant rs4765913 and bipolar disorder. They also showed, together with the Psychiatric Genomic Consortium (PGC), that association was stronger between rs4765913 and a combined sample of schizophrenia and bipolar disorder than with schizophrenia alone [2]. The Schizophrenia Psychiatric Genome-Wide Association Study (GWAS) Consortium [3] conducted a mega-analysis of samples consisting of schizophrenia and bipolar disorder which reached a genome-wide significant association with variant rs4765905. This association was replicated in two studies [4,5].

The Cross-Disorder Group of the Psychiatric Genomics Consortium [6] performed GWAS for samples of schizophrenia, bipolar disorder, ASD, attention deficit-hyperactivity disorder (ADHD), and major depressive disorder (MDD). Variant rs1024582 at *CACNA1C* was associated with cross-disorder at the genome-wide significance level when all five disorders were included. In model selection analyses, variant rs4765914 was associated with sample consisting of bipolar disorder, MDD and schizophrenia [6]. The Schizophrenia Working Group of the Psychiatric Genomics Consortium [7] combined all available schizophrenia samples and identified 108 loci that met genome-wide significance in schizophrenia. One of the loci was at *CACNA1C*, with significant association of variants rs2007044 and rs2239063. Another studies observed genome-wide significant association between variant rs1006737 and schizophrenia [8] and a combined sample of bipolar disorder and schizophrenia [9]. The studies of human brain imaging have suggested that rs1006737 may affect structures and functions of brain in schizophrenia, such as cortical white matter integrity [10–12]. Allelic variation of rs1006737 also has an impact on regional gray matter volume in healthy individuals [13]. Furthermore risk allele AA for schizophrenia increases L-type voltage-gated calcium channel current density and levels of *CACNA1C* mRNA in induced human neurons [14].

Studies in adults have resolved that variants in *CACNA1C* are associated with sleep disorder and sleep traits, including narcolepsy [15], sleep latency [16], and sleep quality [16,17]. Most of the associated variants are located in the third intron of *CACNA1C*, near the seven variants related to psychiatric traits. None of these sleep related variants met the genome-wide significance level. Cacna1c has been found to modulate the electrophysiological architecture of sleep in mice. In a study by Kumar and colleagues [18], haploinsufficiency of Cacna1c reduced EEG spectral gamma power during wake and REM sleep, which might indicate lowered alertness during wakefulness and reduced cortical activation during REM sleep [18]. In addition heterozygous Cacna1c mice demonstrated lower REM sleep rebound after sleep deprivation [18]. Positive symptoms of schizophrenia are related to phenomenological and neurobiological features of REM sleep [19]. The malfunctioning REM-sleep processes of heterozygous *CACNA1C* knockout mice resemble the impaired sleep regulation observed in schizophrenia [18].

Sleep disturbances are common in bipolar disorder [20] and schizophrenia [21]. In children and adolescents, sleep problems are highly-prevalent in tandem with psychiatric disorders, such as ADHD, anxiety, mood disorders, and ASD [22]. Insufficient sleep has negative impact on infant´s neurobehavioral and cognitive functions and health [23]. Short sleep duration, prolonged sleep onset and frequent night awakenings are associated with social-emotional problems in toddlers [24]. There are strong genetic influences for consolidation of sleep in infancy [25], but little is known regarding how genetic variation affects sleep in early childhood. Touchette et al. 2013 reported that nighttime sleep duration is strongly influenced by genetic factors [25]. Their study comprised 995 twins and sleep duration was measured at 6, 18, 30 and 48 months of age. They observed strong genetic influences at consolidated night time sleep duration at 6, 30 and 48 months. In contrast shared environmental influences explained a larger proportion of variance in day time sleep duration [25]. First GWAS for sleep duration in children detected genome-wide significant association at chromosome 11 but this was not replicated in independent samples [26]. Children were 2–14 years old. No GWAS for sleep traits in infants has been published yet.

In this study we attempted to determine whether variants in *CACNA1C* are associated with altered sleep parameters in Finnish infants. We were interested in this question because 1) variants in this gene have been associated with psychiatric disease, especially bipolar disorder and schizophrenia, 2) bipolar disorder and schizophrenia are associated with various sleep disturbances, 3) the Cacna1c channel has been implicated in sleep-wake regulation, and 4) there are strong genetic influences for sleep consolidation in infancy. We explored the data from 1089 Finnish infants that underwent genome wide genotyping and from which infant sleep questionnaire data was available. A small subset of these babies had also undergone polysomnography. To assess insufficient and disturbed sleep we examined sleep duration, nightly awakenings, sleep latency, WASO, SEI and REM/NREM ratio in these infants. We chose to evaluate for seven gene variants that have been shown to be associated genome-wide significantly with bipolar disorder and schizophrenia. The primary finding was that three of the SNPs were associated with prolonged sleep latency in a statistically significant manner. None were associated with changes in total sleep time, or nighttime awakenings.

## Materials and methods

The study sample, CHILD-SLEEP [27], is a Finnish birth cohort collected from Pirkanmaa Hospital District, comprising 1643 infants born April 2011−February 2013, and their parents. The focus of CHILD-SLEEP is on the role of early sleep and circadian rhythm in general population. In this study, the parental questionnaire data related to the sleep of children and successfully genotyped DNA samples were available from 1086 babies (520 girls and 566 boys) who were 8 months old. The ethical approval for CHILD-SLEEP was obtained from the Ethical Committee of Pirkanmaa Hospital District (R11032/9.3.2011). The written informed consents were obtained from the parents. Families were informed of their rights to terminate their participation in the study at any time during data collection.

The evaluation of sleep of the infants in this study is based on parental questionnaires ISQ (the infant sleep questionnaire) and BISQ (a brief screening questionnaire for infant sleep problems) [28,29]. ISQ and BISQ are both reliable and valid measures of infant sleep [30].The selected phenotypes for this study were total sleep time (TST), the number of night awakenings and sleep latency at the age of eight months. To assess these sleep patterns of the infants their parents were asked the following questions: “How many hours does your child sleep at night (19 pm - 07am)?” (Sleep duration at night, BISQ10) and “How many hours does your child sleep at daytime (07am– 19pm)?” (Sleep duration at daytime, BISQ11). BISQ10 and BISQ11 were aggregated for total sleep time. The information for night awakenings (ISQ5) was collected with a question “How many times a night (between 24.00 and 06.00), your baby will usually wake up and need reassurance?” (0 = Does not wake up at all, 1 = Once a night, 2 = Two times at night, 3 = Three times at night, 4 = Four times at night, 5 = Five or more times at night). The question for Sleep latency (ISQ1) was “How long does it usually take to settle your baby to sleep?” (1 = Less than 10 minutes, 2 = 10–20 minutes, 3 = 20–30 minutes, 4 = 30–40 minutes, 5 = 40–50 minutes, 6 = 50–60 minutes, 7 = one hour or more). Total sleep time and night awakenings were normally distributed. Because sleep latency for babies was skewed, we dichotomized the variable based on the mean of the sleep latency in this study (Table 1) and earlier sleep studies in children [24,31] as less than 20 minutes and more than 20 minutes.

**Table 1:** Description on study variables.

Variable	N	Range	Mean	P^a^
TST	1086	9–17 h	13.3 h	0.67
Night awakenings	1086	0 - ≤ 5 times	2 times at night	0.64
Sleep latency	1086	<10 min - ≤ 1 h	10–20 min	0.17
WASO	63	24–120 min	67.2 min	0.09
SEI	63	76–96%	86.7%	0.62
R_NREM	63	0.30–0.73	0.49	0.16
Family atmosphere^b^	1086	7–43	13.5	0.81
Breastfeeding	1086	-	44.8% (breastfed)	0.96
Illness	1086	-	12.4% (had illness)	0.82

^a^p-value of t-test for the difference between boys and girls

^b^Small score reflects better atmosphere

A subcohort of the children Child Sleep babies underwent an ambulatory over-night PSG in three different ages (1, 8, and 24 months of age) as described in detail earlier [32]. At the age of eight months, 72 infants were recorded. The recordings were started at the families’ homes close to the babies’ usual bed time. However, the objective sleep latency could not be measured in the PSGs, as the evening routines varied substantially. Often babies fell asleep while eating and the real “lights off” time could not be defined. The length of the recordings varied depending on the habitual length of over-night sleep of the babies.

For the purpose of this study, nine hours of recording were analyzed, beginning at the sleep onset. The sleep stages were scored according to the established rules [33]. The sleep parameters chosen for this study were WASO (wake after sleep onset), SEI (sleep efficiency) and R/NREM (ratio between REM and NREM sleep) (Table 1). WASO was the time of wakefulness (in minutes) during the nine hours of recording. SEI was the total sleep time divided by the nine hours of recording. R/NREM ratio was the amount of stage R sleep divided by the amount of NREM sleep during the nine hours [32].

Umbilical cord blood sample was drawn from each newborn. DNA was extracted according to standard procedures. DNA samples were genotyped with Illumina Infinium PsychArray BeadChip at Estonian Genome Centre and quality control (QC) was performed with PLINK (http://pngu.mgh.harvard.edu/~purcell/plink/). Markers were removed for missingness (>5%), Hardy-Weinberg equilibrium (p-value < 1 x 10^−6^), and low minor allele frequency (< 0.01). Individuals were checked for missing genotypes (>5%), relatedness (identical by descent calculation, PI_HAT>0.2) and population stratification (multidimensional scaling). After QC, phenotype and high quality genotype data at 8 months was available for 1086 (parental questionnaires) and 63 (PSG) babies. Genotyped data was imputed with IMPUTEv2 [34] against Finnish WGS (whole-genome sequencing) [35] and 1000 genomes [36] (Phase 3, released at February, 2013) reference panels.

Seven SNPs (single nucleotide polymorphism) earlier associated to psychiatric traits at *CACNA1C* with genome wide significance were selected for analyses (Table 2). The association between seven SNPs and quantitative sleep measures were tested with linear (TST and nightly awakenings) and logistic (sleep latency) regression analyses implemented with PLINK. Covariates used in the analyses were gender, first three principals components of GWAS, family atmosphere [27], breastfeeding (dichotomized variable: breastfeeding/breastfeeding + formula or formula), and babies’ illnesses (milk allergy, other allergy, colic, infections, reflux, congenital heart disease, innate developmental disorder, neurological disorder, growth retardation, other illness). Babies´ illnesses were used as a dichtomized variable: no illness / 1 or more illness. Descriptions of the study variables are presented in Table 1. Associations were corrected for multiple testing by the max (T) permutation in PLINK with 10 000 permutations per SNP. The criterion for significance was set a permutation p < 0.05. Because of multiple correlated phenotypes Bonferroni correction was not used in this study. It is likely to be overly conservative as the analyses are not independent of each other.

**Table 2:** SNPs associated with psychiatric traits in genome-wide significant level in *CACNA1C*.

SNP	Phenotype	Reference
rs2007044	Schizophrenia	[7]
rs1006737	bipolar disorder, schizophrenia	[8,9]
rs4765905	bipolar disorder, Schizophrenia	[3–5]
rs1024582	bipolar disorder, schizophrenia	[6]
rs4765913	bipolar disorder, schizophrenia	[2]
rs4765914	bipolar disorder, MDD, schizophrenia	[6]
rs2239063	Schizophrenia	[7]

Power calculations were performed with the Genetic Power Calculator (http://pngu.mgh.harvard.edu/~purcell/gpc/) [37] assuming an additive model (TST and night awakenings) or case-control for threshold-selected quantitative trait (sleep latency), 1% quantitative trait locus (QTL) variance or additive genetic variance and perfect linkage disequilibrium between QTL and the markers. There was 85% statistical power to detect variants with significance level of 0.05 when polymorphisms with allele frequencies from 0.2 to 0.3 were analyzed.

## Results

**Fig 1:**
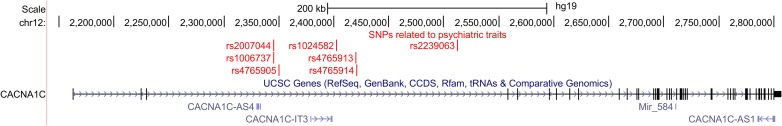
Location of studied SNPs at *CACNA1C*. Seven SNPs associated with psychiatric disorders are located in intron three of *CACNA1C* gene.

**Fig 2:**
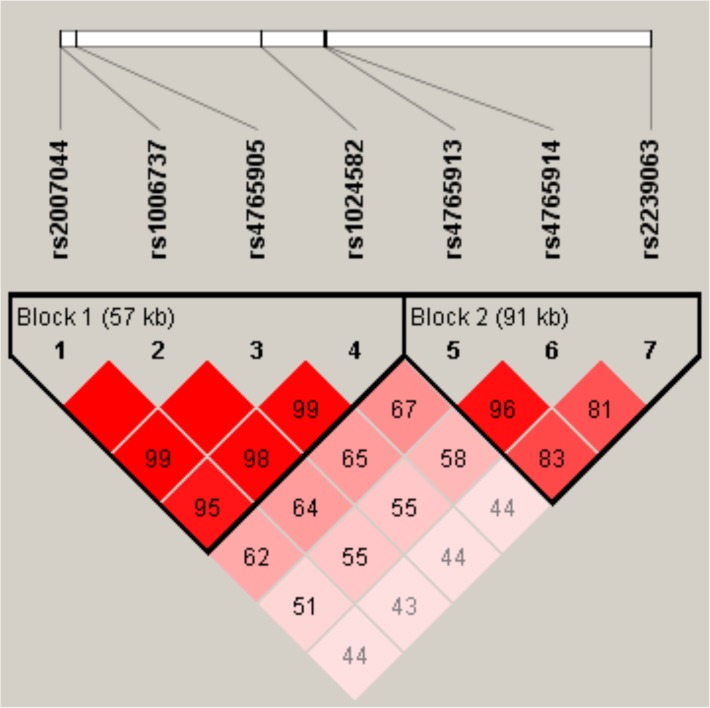
LD structure of the SNPs analyzed at *CACNA1C*. Markers with linkage disequilibrium (0<r^2^≤1) are shown in red through pale light pink (color intensity decreases with decreasing r^2^ value).

All seven variants included in the analyses of this study lie within 167 kb region of the *CACNA1C* intron three (Fig 1). LD-structure of the studied variants is presented in Fig 2. There are two LD-blocks in the region. The first region comprises the variants rs2007044, rs1006737, rs4765905, and rs1024582 (D`≥ 0.95). Another LD-block consists of SNPs rs4765913, rs4765914, and rs2239063 (D`≥ 0.81).

The results of the association analyses are shown in Table 3. Beta values indicate the direction of the effect for the minor allele. Empirical p-value 1 (EMP1) is a point-wise p-value from 10 000 permutations and empirical p-value 2 (EMP2) means corrected empirical p-value over all studied SNPs. In the primary analyses we used only sex as a covariate. Variants rs4765913 (minor allele A), rs4765914 (minor allele T), and rs2239063 (major allele A) were associated with longer sleep latency (permuted P < .05, EMP1) in additive model. When permuted 10 000 times over all 7 variants (EMP2) the variants rs4765914 and rs2239063 remained significant. In dominant model the strongest association was detected between variant rs2239063 and sleep latency (beta = −0.4023, P = 0.0037, EMP1 = 0.0036, EMP2 = 0.017). In recessive model variant rs4765913 showed the strongest association for sleep latency (beta = 0.8076, P = 0.003487, EMP1 = 0.0025, EMP2 = 0.0167). There was no significant association between studied variants and night awakenings or sleep duration. When the covariates family atmosphere, breastfeeding, babies’ illnesses and genetic principal components were added to the analyses, the results for sleep latency remained significant.

**Table 3:** The results of association analyses between *CACNA1C* variants and TST, night awakenings and sleep latency, N = 1086.

SNP	TST				Night awakenings				Sleep latency			
	beta	P	EMP1^a^	EMP2^b^	beta	P	EMP1^a^	EMP2^b^	beta	P	EMP1^a^	EMP2^b^
rs2007044*	-0.02258	0.6871	0.6825	0.9935	-0.04966	0.4547	0.4558	0.8971	0.06581	0.5299	0.5212	0.9486
rs1006737	-0.02226	0.6996	0.7002	0.9945	-0.07104	0.3011	0.2945	0.733	0.05984	0.5821	0.5683	0.9696
rs4765905	-0.02166	0.7092	0.7135	0.9952	-0.05912	0.3922	0.3802	0.8402	0.04947	0.6511	0.6379	0.9863
rs1024582	-0.01756	0.7545	0.7571	0.9983	-0.03266	0.6229	0.6201	0.98	0.02755	0.7936	0.787	0.9993
rs4765913	-0.04492	0.4643	0.4686	0.9069	0.04481	0.5405	0.5384	0.951	0.2561	**0.02332**	**0.0237**	0.08759
rs4765914	-0.05098	0.4365	0.4339	0.8845	0.1299	0.09763	0.09774	0.324	0.3026	**0.01115**	**0.0106**	**0.0423**
rs2239063*	-0.01161	0.8368	0.8384	0.9998	0.02467	0.7116	0.7183	0.9943	-0.2767	**0.01138**	**0.011**	**0.0426**

Additive model, adjusted with sex

*imputed

^a^Empirical p-value 1 (EMP1) = point-wise p-value from 10,000 permutations

^b^Empirical p-value 2 (EMP2) = corrected empirical p-value by max (T) permutations, TST = total sleep time.

When girls and boys were analyzed separately, association of variant rs4765914 with sleep latency was statistically significant in boys (beta = 0.4331, P = 0.008, EMP1 = 0.008, EMP2 = 0.037) but not in girls in additive model. In dominant model rs4765914 (minor allele T) shortened TST in boys (beta = -0.248, P = 0.0275, EMP1 = 0.0309, EMP2 = 0.1162). There was no significant association between sex and *CACNA1C* variants in night awakenings.

We also performed haplotype analyses for sleep latency, TST and night awakenings. They were in line with primary analyses and several haplotypes were associated with sleep latency (P < 0.05). In logistic regression analyses two haplotypes were significant after 10 000 permutation over all seven SNPs (EMP2 < 0.05). Of them frequencies of haplotype (CC) comprising variants rs4765914 and rs2239063 and TCC of variants rs4765913, rs4765914 and rs2239063 differed significantly between infants with short versus long latency (P = 0.001581, P = 00169, respectively). Haplotypes comprising protective alleles for psychiatric disorders were more common in short latency (haplotype CC: freq short/long latency = 0.3156/0.243, haplotype TCC: freq short/long latency = 0.3172/0.245). There was no significant association between any of the haplotypes and TST or night awakenings.

We analyzed the association of polysomnography (PSG) variables in a sample of 63 babies at 8 months of age. There was no significant correlation between subjective measure of nightly awakenings (ISQ5) and objective measures WASO or SEI. The results of PSG analyses are shown in Table 4. There was no significant association between 7 variants and the PSG variables.

**Table 4:** Polysomnography results, N = 63.

SNP	WASO				SEI				REM/NREM			
	beta	P	EMP1^a^	EMP2^b^	beta	P	EMP1^a^	EMP2^b^	beta	P	EMP1^a^	EMP2^b^
rs2007044*	2.304	0.6337	0.6343	0.9726	-0.4188	0.6665	0.6698	0.9829	-0.0001271	0.995	0.9956	1
rs1006737	-3.049	0.5525	0.5487	0.9365	0.5307	0.6072	0.6158	0.9638	-0.004783	0.8249	0.8305	0.9988
rs4765905	-2.874	0.5799	0.5765	0.9505	0.5024	0.6307	0.6395	0.9724	-0.00398	0.8549	0.8549	0.9995
rs1024582	-3.054	0.5241	0.5321	0.9193	0.3914	0.6849	0.6919	0.9867	-0.009561	0.6486	0.6526	0.9754
rs4765913	-1.09	0.8247	0.8245	0.999	0.1462	0.8825	0.8845	0.9997	0.005384	0.8056	0.8096	0.9984
rs4765914	-0.2365	0.9631	0.9644	1	-0.08532	0.9337	0.9304	1	0.008536	0.7147	0.7171	0.9905
rs2239063*	-8.536	0.1379	0.1395	0.3816	1.785	0.1223	0.1252	0.3471	-0.03394	0.1514	0.15	0.4028

WASO = wake after sleep onset, SEI = sleep efficiency, REM = rapid eye movement sleep, NREM = non-rapid eye movement sleep, additive model, adjusted with sex, three first principal components of GWAS, home atmosphere, breastfeeding and babies’ illnesses

*imputed

^a^Empirical p-value 1 (EMP1) = point-wise p-value from 10,000 permutations

^b^Empirical p-value 2 (EMP2) = corrected empirical p-value by max (T) permutations.

## Discussion

In this study, some of the variants related to psychiatric traits showed nominally significant association with sleep. In the analyses of the whole sample variants rs4765913, rs4765914, and rs2239063 were associated with sleep latency. The minor allele A of rs4765913 was associated with longer sleep latency. Psychiatric GWAS Consortium Bipolar Disorder Working Group [2] reported that allele A is associated with bipolar disorder (P = 1.52x10^-8^) and with combined samples of bipolar disorder and schizophrenia (P = 7.7x10^-8^). In our study, the minor allele T of rs4765914 was associated with longer time of sleep latency in infants. Cross-Disorder of the Psychiatric Genomics Consortium [6] reported that this same allele is associated with bipolar disorder, major depressive disorder, and schizophrenia. T allele is also associated with amygdala structure and function in adolescents; in the study of Sumner and colleagues [38] homozygous carriers of T allele exhibited smaller amygdala volume compared to individuals with homozygous major C allele [38]. Reduced amygdala volume has been observed in bipolar disorder in adults and youth in earlier studies [39,40]. There is a complex interplay between sleep and emotions; sleep deprivation impairs the connectivity between amygdala and prefrontal cortex, which have a direct impact on individual´s ability to regulate emotions [41].

Finally, in our study the major allele A of rs2239063 was associated with longer sleep latency in 8-month-old babies. This same allele is associated with schizophrenia at the genome-wide significance level (P = 1.93e^-8^) [7]. Long sleep latency is common in schizophrenia [21] and bipolar disorder [20]. Considering haplotype analyses we observed that frequencies of haplotypes which comprised the protective alleles for psychiatric traits, CC (rs4765914 and rs2239063) and TCC (rs4765913, rs4765914 and rs2239063) were significantly more frequent within babies with short sleep latency time compared to infants with long latency.

When we analyzed boys and girls separately, the psychiatric risk allele T of rs4765914 was significantly associated with prolonged sleep latency and shortened TST in boys but not in girls. In case this finding is not considered as lack of power due to small sample size of studied groups but true biological gender difference, it suggests that male gender may be genetically vulnerable for sleep disturbances. In earlier studies there were sex-specific differences in influences of *CACNA1C* variants on mood disorders [42] and functional recovery from episodes of schizophrenia [43]. This could be explained by hormonal differences. Estrogen directly potentiates neuronal L-type Ca^2+^ channels [44] and inhibits Ca^2+^ influx through L-type voltage-gated CA^2+^ channels [45].

Variants in *CACNA1C* are associated with sleep latency and quality of sleep in adults [16,17]. In our study we observed that some of the *CACNA1C* variants for psychiatric disorders were found to be associated with sleep latency in babies at 8 months-of-age. These variants were not significantly associated with sleep duration or nightly awakenings. Our results for sleep duration in infants are in line with earlier studies where no genome-wide significant association for sleep duration at *CACNA1C* has been reported [16,26,46–50]. GWAS for nightly awakenings has not been published in adults. More studies are needed to see if *CACNA1C* regulates nightly awakenings.

The limitations of this study were the cross-sectional approach of the research and the fact that we lacked replication data. Further, the power to detect risk variants for sleep disturbances with polysomnography was limited because the amount of objective data was quite small. The objective sleep latency of the infants could not be measured because the recording was not started at bedtime. This is why we chose parental questionnaires as a measure for the sleep latency.

There are also several other candidate genes for sleep disturbances like recently detected *RBFOX3* in meta-analyses of sleep latency with large dataset [51]. In our study we focused *CACNA1C* because of its central role in sleep and psychiatric problems. Power for GWAS was limited because the sample size was relatively small but meta-analyses with other childhood cohorts will be performed later. The results of other candidate genes for sleep disturbances in CHILD-SLEEP project will be reported elsewhere.

Nonetheless, overall C*ACNA1C* remains a potential candidate gene for sleep disturbances and the variants may affect sleep already in early childhood. Collection of CHILD-SLEEP data is continuing and we will follow the sleep and mental health of these children. Understanding how the genetic variants influence in sleep/wake regulation already in infancy may have an impact on helping the families to interfere in infants´ sleep problems at early stage. In a long term the growing knowledge of genetics could also be used as basis of development of therapies and early diagnostics for sleep and neuropsychological disorders.
